# Supplementary material for: Citrullination of pyruvate kinase M2 by PADI1 and PADI3 regulates glycolysis and cancer cell proliferation
Source: Nat Commun. 2021 Mar 19;12:1718. doi: 10.1038/s41467-021-21960-4 (PMC7979715; doi:10.1038/s41467-021-21960-4)
Supplement: Supplementary file 1 — Supplementary information [file 41467_2021_21960_MOESM1_ESM.pdf]

Supplementary Figures and Legends.

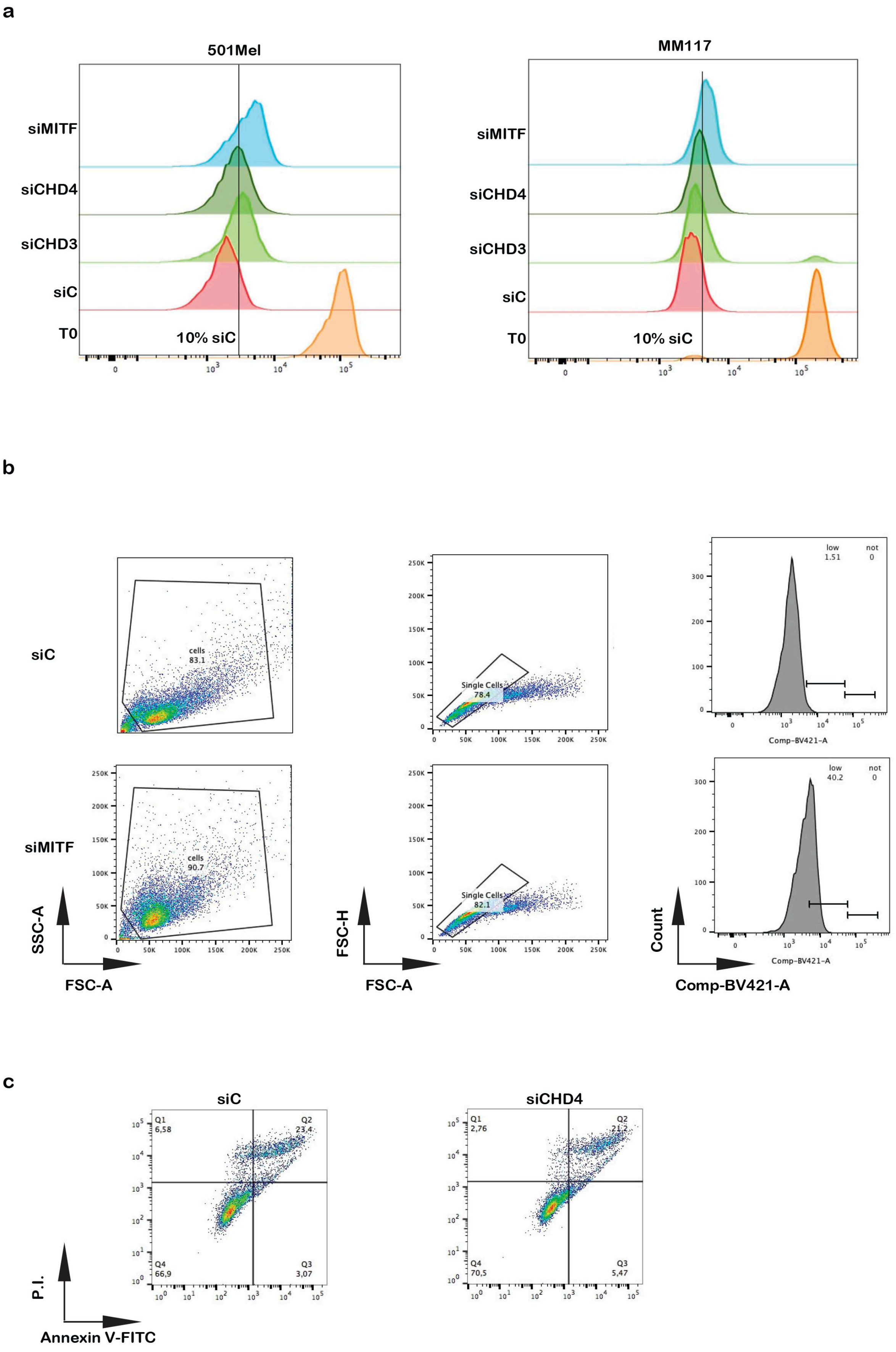

Coassolo et al., Supplementary Figure 1

Supplementary Figure 1. a. CHD4 silencing leads to lowered cell growth. Examples of Brilliant Violet 421 flow cytometry profiles using Cell Trace Violet incorporation to measure proliferation. Flow cytometry profiles are shown for 501Mel or MM117 cells transfected with control siRNA or siRNA to silence CHD3 and CHD4 or MITF. T0 indicates the profile of cells labelled just before flow cytometry, where no dilution of the dye is seen. The vertical line shows the cutoff used to designate 10% of the siC cells. All cells to the right of the line in the other conditions are designated slow-proliferating. **b**. Example of gating strategy using siC and siMITF transfected cells. After harvesting, cells were first gated by FSC-A/SSC-A to separate live cells from dead cells and debris. Live cell fractions (80-95%) were then gated FSC-A/FSC-H to identify single cells and remove doublets with abnormal FSC-A/FSC-H ratio. Gated single cell fluorescence of Brilliant Violet 421 was then assessed and quantified. **c**. For apoptosis, cells were gated using Propidium Iodide and Annexin V-FITC.

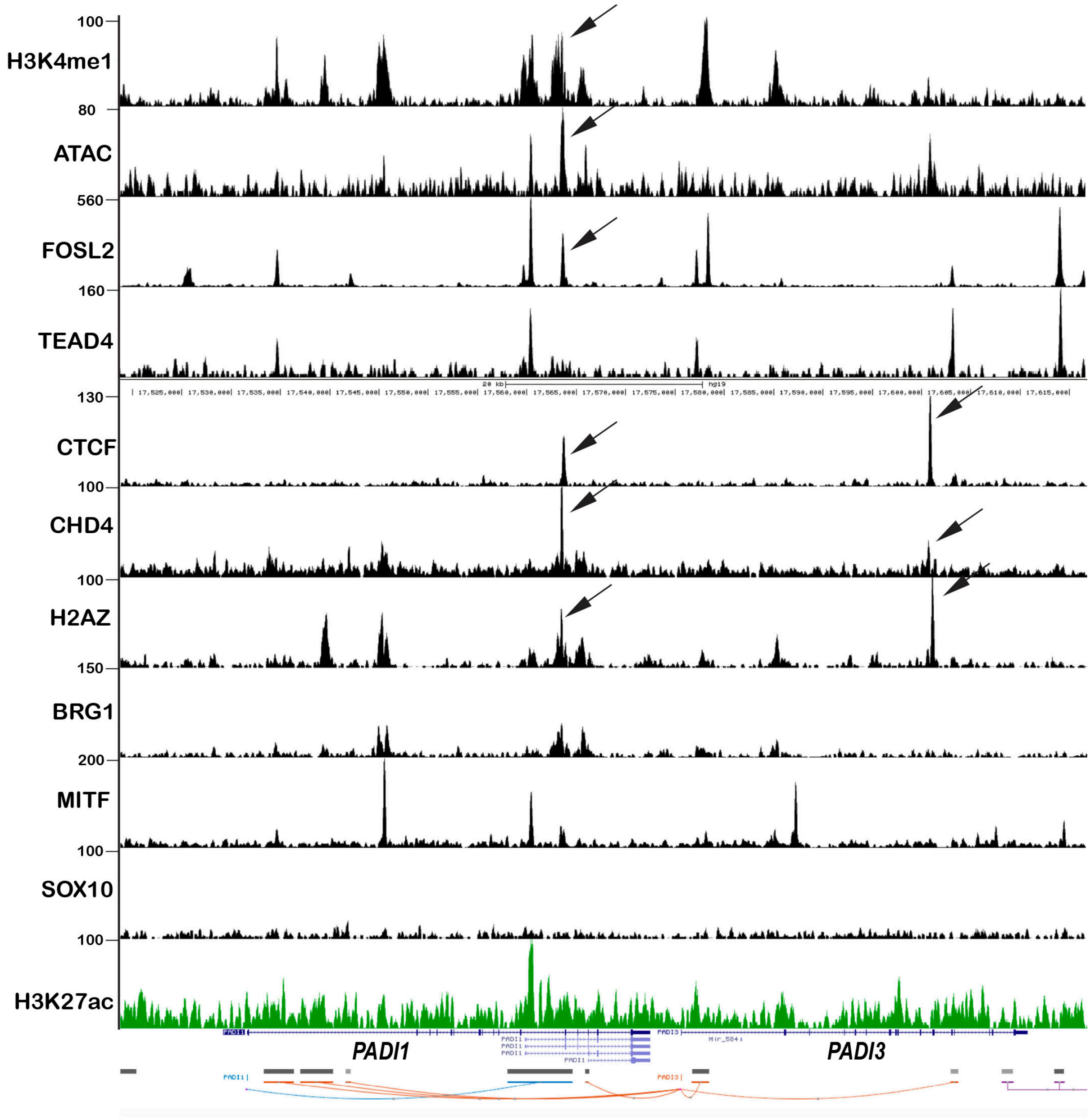

Coassolo et al., Supplementary Figure 2

Supplementary Figure 2. Regulation of the *PADI1-PADI3* locus. CHD4, CTCF and FOSL2 co-occupy a regulatory element at the *PADI1-PADI3* locus. Screenshot of UCSC genome browser at the *PADI1-PADI3* locus showing the indicated ChIP-seq data. Arrows highlight the putative cis-regulatory elements occupied by CTCF, FOSL1 and CHD4 and marked by ATAC-seq, H3K4me1, BRG1 and H2AZ. The following data sets were used: H3K4me1 GSM2476344; ATAC GSM2476338; FOSL2 GSM2842801; TEAD4 GSM2842802 [1]; CHD4 this study. Other data are from Laurette et al., 2015 [2].

https://www.ncbi.nlm.nih.gov/geo/query/acc.cgi?acc=GSE94488

https://www.ncbi.nlm.nih.gov/gds/?term=GSM2476338[Accession]

https://www.ncbi.nlm.nih.gov/geo/query/acc.cgi?acc=GSE94488

https://www.ncbi.nlm.nih.gov/geo/query/acc.cgi?acc=GSM2842802

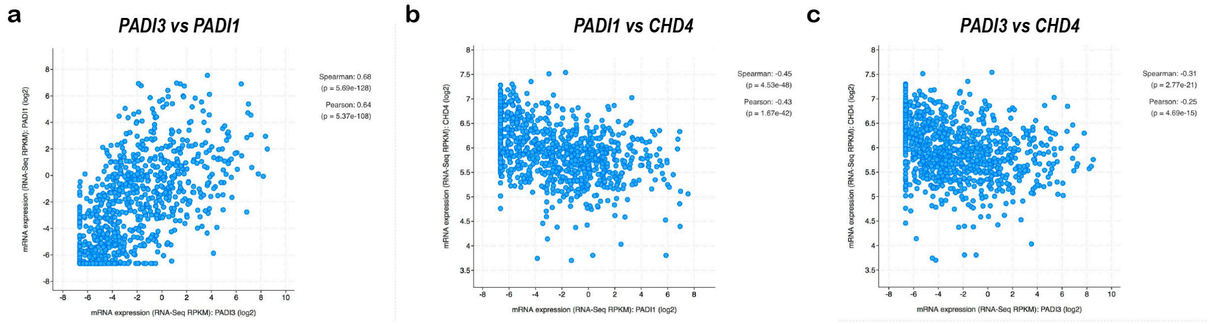

Coassolo et al., Supplementary Figure 3

Supplementary Figure 3. a-c. Analyses of gene expression in the Cancer Cell-Line Encyclopedia. Each graph shows the correlation of expression between the indicated genes with their Spearman and Pearson coefficients and p-values. Expression of PADI1 and PADI3 correlated positively, while expression of PADI1 or PADI3 correlated negatively with CHD4.

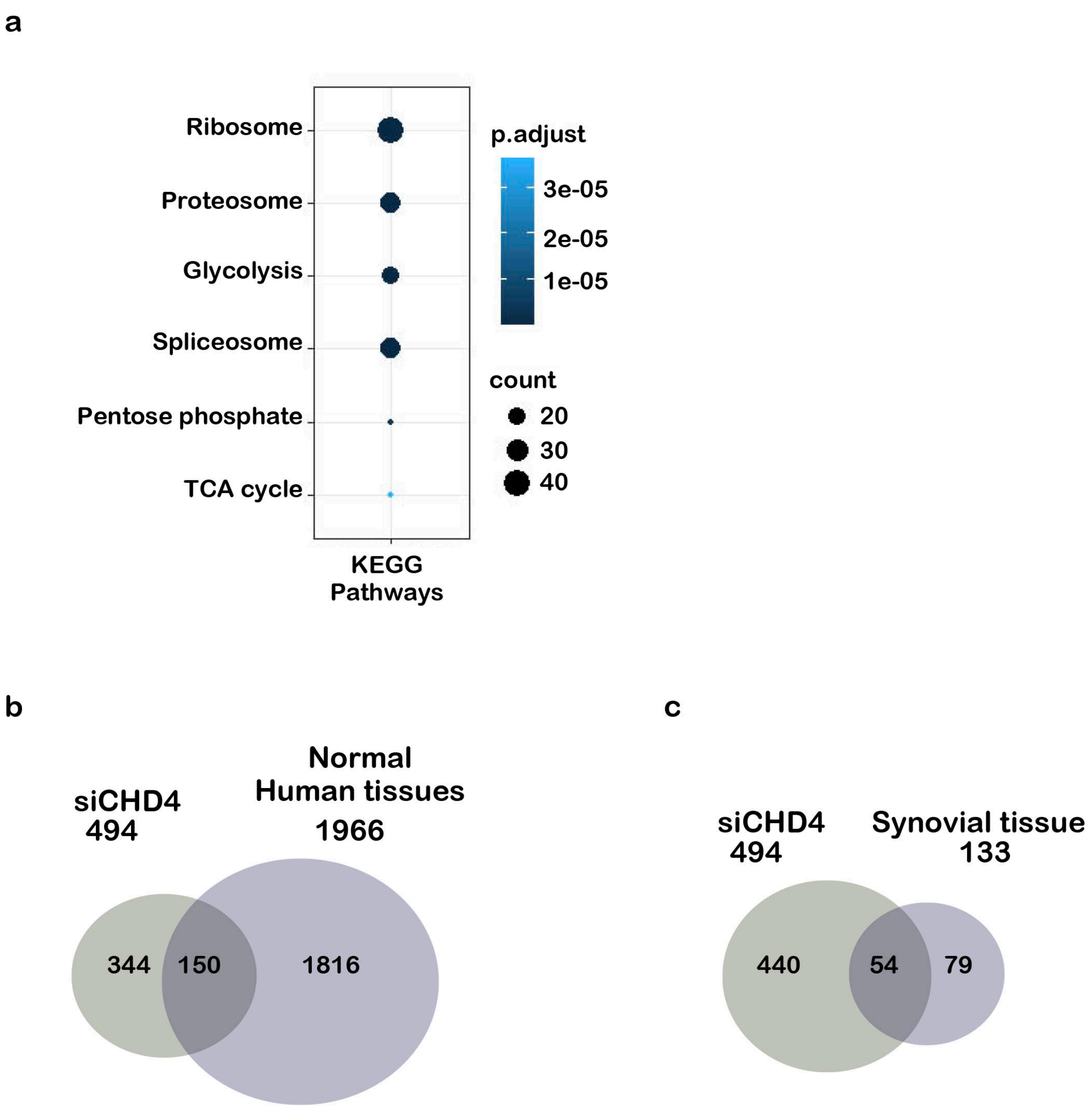

Coassolo et al., Supplementary Figure 4

Supplementary Figure 4. Proteins showing enhanced citrullination after siCHD4 silencing. **a**. Bubble plot showing the KEGG ontology terms of the proteins showing enhanced citrullination in the siCHD4 cells. **b-c**. Venn diagrams showing overlap between the proteins enriched in the siCHD4 extracts and previously identified proteins in normal human tissues or synovial tissue.

a

PKM2 Peptide	C>R
TATESFASDPILYR*PVAVAL	R106
FGVEQDVDMVFASFIR*KAS	R246
IENHEGVRR*FDEILEASDGI	R279
DPVQEAWAEDVDLR*VNFA	R489

b

Sequence: TATESFASDPILYRPVAVALDTK, R14-Deamidated (0.98402 Da)
Charge: +3, Monoisotopic m/z: 822.68704 Da (-76.62 mmu/-93.13 ppm), MH+: 2466.04657 Da, RT: 77.4246 min,
Identified with: Sequest HT (v1.17); XCorr:3.44, Percolator q-Value:3.6e-4, Percolator PEP:7.3e-4, ptmRS: Best Site Probabilities: R14(Deamidated): 100,

#1	b	b²	Seq.	y	y²	#2
1	102.1	51.5	T			23
2	173.1	87.0	A	2365.2	1183.1	22
3	274.1	137.6	T	2294.2	1147.6	21
4	403.2	202.1	E	2193.1	1097.1	20
5	490.2	245.6	S	2064.1	1032.6	19
6	637.3	319.1	F	1977.1	989.0	18
7	708.3	354.7	A	1830.0	915.5	17
8	795.4	398.2	S	1759.0	880.0	16
9	910.4	455.7	D	1671.9	836.5	15
10	1007.4	504.2	P	1556.9	779.0	14
11	1120.5	560.8	I	1459.9	730.4	13
12	1233.6	617.3	L	1346.8	673.9	12
13	1396.7	698.8	Y	1233.7	617.3	11
14	1553.7	777.4	R-deam	1070.6	535.8	10
15	1650.8	825.9	P	913.5	457.3	9
16	1749.9	875.4	V	816.5	408.7	8
17	1820.9	911.0	A	717.4	359.2	7
18	1920.0	960.5	V	646.4	323.7	6
19	1991.0	996.0	A	547.3	274.2	5
20	2104.1	1052.6	L	476.3	238.6	4
21	2219.1	1110.1	D	363.2	182.1	3
22	2320.2	1160.6	T	248.2	124.6	2
23			K	147.1	74.1	1

c

Sequence: FGVEQDVDMVFASFIR, R16-Deamidated (0.98402 Da)
Charge: +2, Monoisotopic m/z: 931.24534 Da (+300.13 mmu/+322.28 ppm), MH+: 1861.48340 Da, RT: 106.5405 min,
Identified with: Sequest HT (v1.17); XCorr:4.54, Percolator q-Value:4.1e-4, Percolator PEP:1.9e-4, ptmRS: Best Site Probabilities:R16(Deamidated): 100, Fragment match tolerance used for search: 0.6 Da Fragments used for search: $-H_2O$; y; $-NH_3$; y; b; b; $-H_2O$; b; $-NH_3$; y Proteins (1):
- Pyruvate kinase PKM OS=Homo sapiens OX=9606 GN=PKM PE=1 SV=4

#1	b	Seq.	y	#2
1	148.1	F		16
2	205.1	G	1712.8	15
3	304.2	V	1655.8	14
4	433.2	E	1556.7	13
5	561.3	Q	1427.7	12
6	676.3	D	1299.6	11
7	775.4	V	1184.6	10
8	890.4	D	1085.5	9
9	1021.4	M	970.5	8
10	1120.5	V	839.5	7
11	1267.6	F	740.4	6
12	1338.6	A	593.3	5
13	1425.6	S	522.3	4
14	1572.7	F	435.3	3
15	1685.8	I	288.2	2
16		R-deam.	175.1	1

d

Sequence: RFDEILEASDGIMVAR, R1-Deamidated (0.98402 Da)
Charge: +3, Monoisotopic m/z: 608.67131 Da (+366.96 mmu/+602.89 ppm), MH+: 1823.99938 Da, RT: 72.3455 min,
Identified with: Sequest HT (v1.17); XCorr:4.47, Percolator q-Value:1.4e-3, Percolator PEP:1.7e-2, ptmRS: Best Site Probabilities:R1(Deamidated): 100, Fragment match tolerance used for search: 0.6 Da Fragments used for search: $-H_2O$; y; $-NH_3$; y; b; b; $-H_2O$; y
Proteins (1): - Pyruvate kinase PKM OS=Homo sapiens OX=9606 GN=PKM PE=1 SV=4

#1	b	b²	Seq.	y	y²	#2
1	158.1	79.5	R-Deam.			16
2	305.2	153.1	F	1665.8	833.4	15
3	420.2	210.6	D	1518.7	759.9	14
4	549.2	275.1	E	1403.7	702.4	13
5	662.3	331.7	I	1274.7	637.8	12
6	775.4	388.2	L	1161.6	581.3	11
7	904.4	452.7	E	1048.5	524.8	10
8	975.5	488.2	A	919.5	460.2	9
9	1062.5	531.8	S	848.4	424.7	8
10	1177.5	589.3	D	761.4	381.2	7
11	1234.6	617.8	G	646.4	323.7	6
12	1347.6	674.3	I	589.3	295.2	5
13	1478.7	739.8	M	476.3	238.6	4
14	1577.8	789.4	V	345.2	173.1	3
15	1648.8	824.9	A	246.2	123.6	2
16			R	175.1	88.1	1

e

Sequence: DPVQEAWAEDVDLR, R14-Deamidated (0.98402 Da)
Charge: +2, Monoisotopic m/z: 822.84120 Da (+460.73 mmu/+559.92 ppm), MH+: 1644.67513 Da, RT: 75.5411 min,
Identified with: Sequest HT (v1.17); XCorr:3.94, Percolator q-Value:3.3e-4, Percolator PEP:8.5e-4, ptmRS: Best Site Probabilities:R14(Deamidated): 100, Fragment match tolerance used for search: 0.6 Da Fragments used for search: $-H_2O$; y; $-NH_3$; y; b; b; $-H_2O$; b; $-NH_3$; y
Proteins (1): - Pyruvate kinase PKM OS=Homo sapiens OX=9606 GN=PKM PE=1 SV=4

#1	b	Seq.	y	#2
1.0	116.0	D		14.0
2.0	213.1	P	1528.7	13.0
3.0	312.2	V	1431.7	12.0
4.0	440.2	Q	1332.6	11.0
5.0	569.3	E	1204.5	10.0
6.0	640.3	A	1075.5	9.0
7.0	826.4	W	1004.5	8.0
8.0	897.4	A	818.4	7.0
9.0	1026.5	E	747.4	6.0
10.0	1141.5	D	618.3	5.0
11.0	1240.5	V	503.3	4.0
12.0	1355.6	D	404.2	3.0
13.0	1468.7	L	289.2	2.0
14.0		R-Deam.	176.1	1.0

Coassolo et al., Supplementary Figure 5

Supplementary Figure 5. Citrullinated arginines in PKM2. **a.** Peptide sequences around the arginine residues that are subject to citrullination. **b-e**. Data from the MS/MS analysis that identifies the modified arginines. Note that citrullination of R246 and R489 located at the C-terminus of the tryptic peptides cannot be unambiguously assigned from this analysis. Confirmation of R246 citrullination is based on previous independent studies and the use of the specific anti-citrulline antibody.

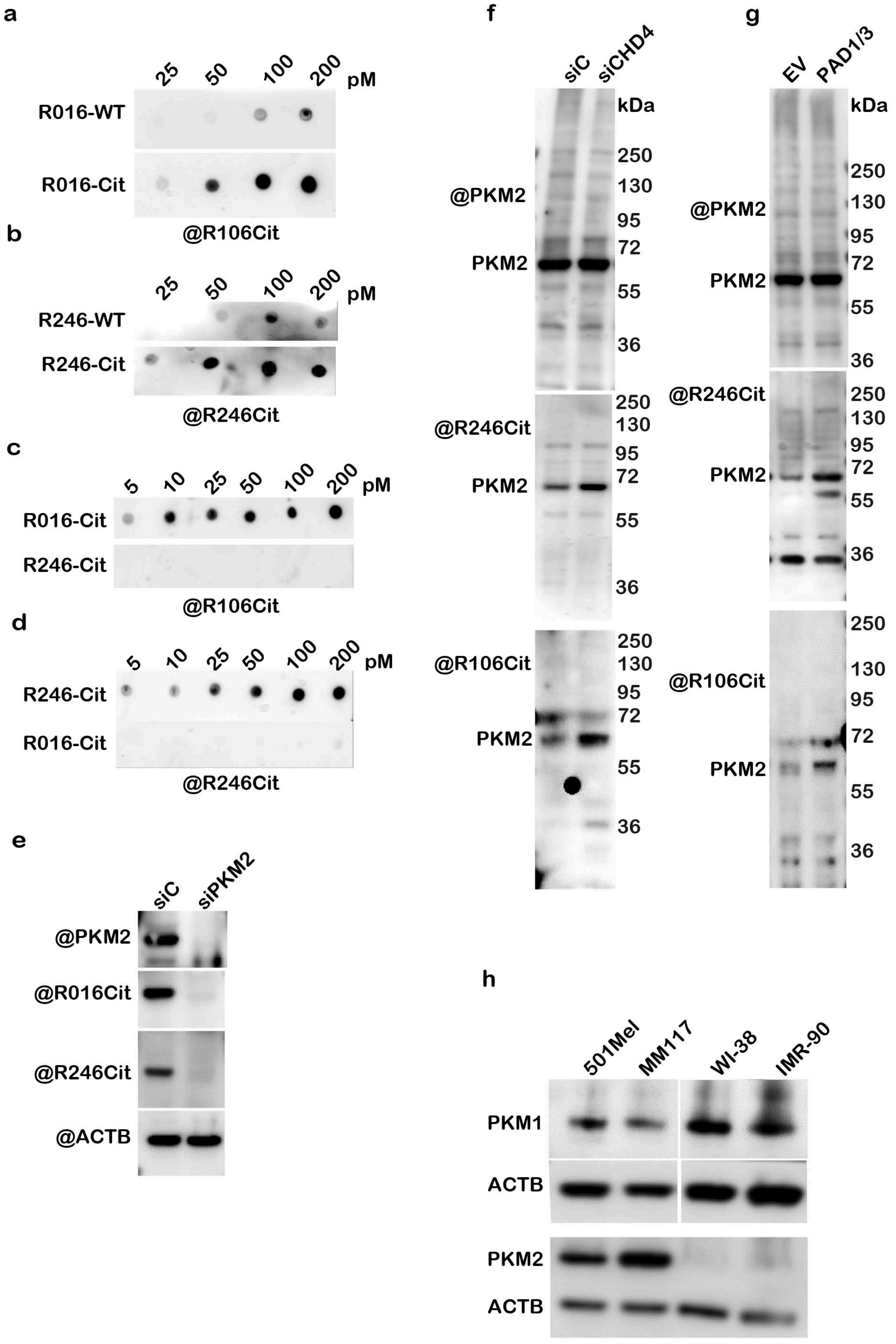

Coassolo et al., Supplementary Figure 6

Supplementary Figure 6. Enhanced citrullination of R106 and R246 following CHD4 silencing or ectopic PAD1 and PAD3 expression. **a-d**. Dot blots with the indicated amounts of wild-type peptides or equivalent peptides where R106 or R246 were replaced by citrulline. **e**. Immunoblots showing that the signals seen with the anti-citrulline antibodies are lost in extracts from cells where PKM2 has been silenced. **f-g**. Immunoblots of cells transfected with the indicated siRNA or vectors. Cell extracts were loaded on three different gels and detected with commercial antibody against PKM2 to ensure equivalent quantities of PKM2 and with antibodies directed against the indicated citrullinated peptides. M shows the migration of the molecular mass standards. **h**. Immunoblots showing expression of PKM1 and PKM2 in the indicated cell extracts as detected with antibodies specific for each isoform.

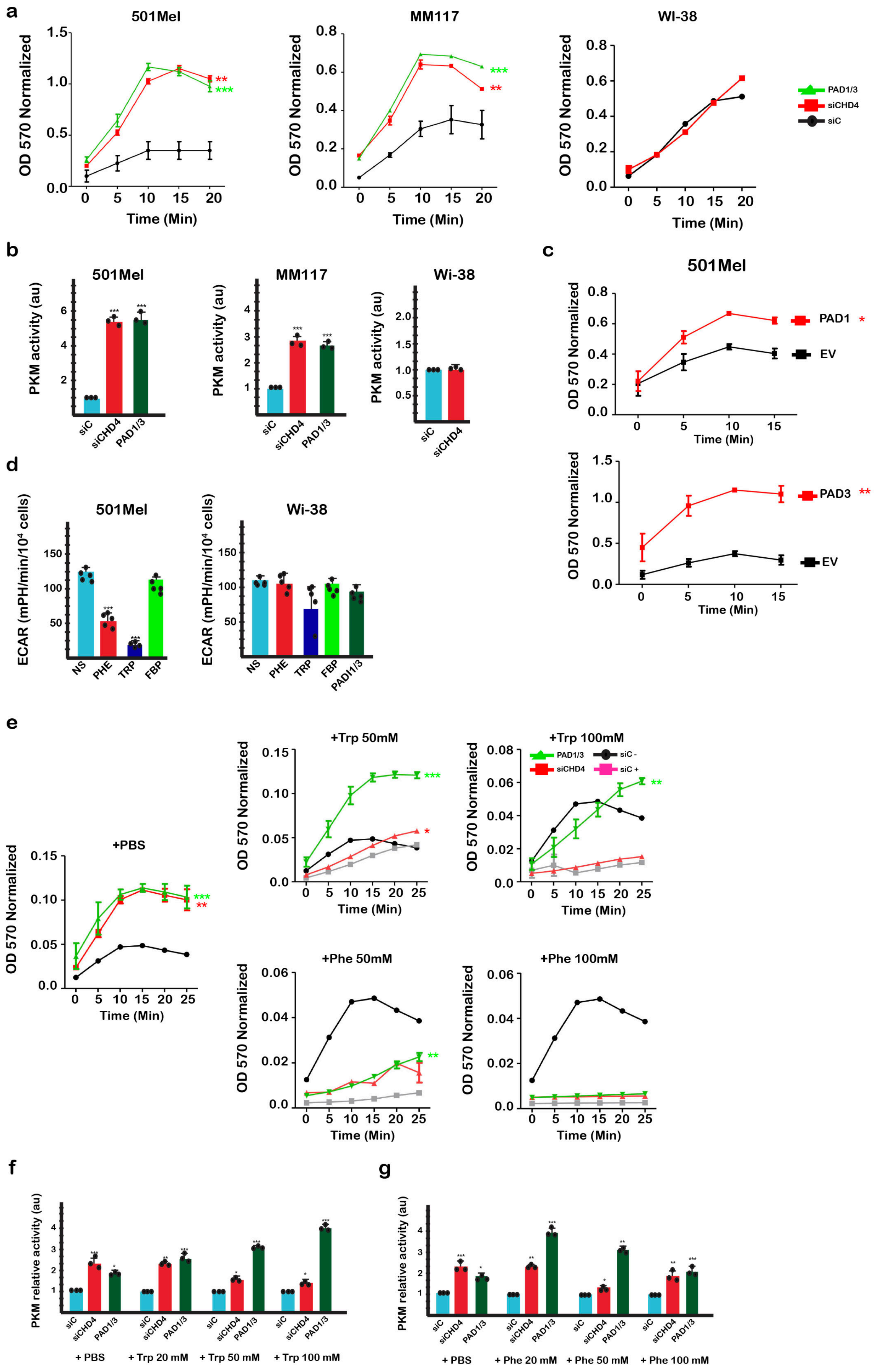

Coassolo et al., Supplementary Figure 7

Supplementary Figure 7. Citrullination stimulates PKM2 enzymatic activity. **a-c.** Measurement of PKM2 or PKM1 (WI-38-cells) enzymatic activity in extracts from cells transfected with the indicated siRNAs or expression vectors. **d**. Glycolysis in 501Mel or WI-38 cells treated with 50 mM of the indicated ligands or transfection of the PAD1/3 expression vectors. N= 3 biological replicates and unpaired t-test with two tailed P-value analyses and confidence interval 95% were performed by Prism 5. P-values: *= $p<0,05$; **= $p<0,01$; ***= *p<0,001*. Data are mean ± SEM. **e-g**. Measurement of PKM2 enzymatic activity in extracts from cells transfected with the indicated siRNAs or expression vectors in presence or absence of the indicated concentrations of exogenous Phe or Trp or PBS as control. N= 3 or 4 biological replicates as indicted by the data points in each panel. Values for PKM2 enzymatic activity were determined by Prism 5 using a 2-way ANOVA test. P-values: *= $p<0,05$; **= $p<0,01$; ***= $p<0,001$.

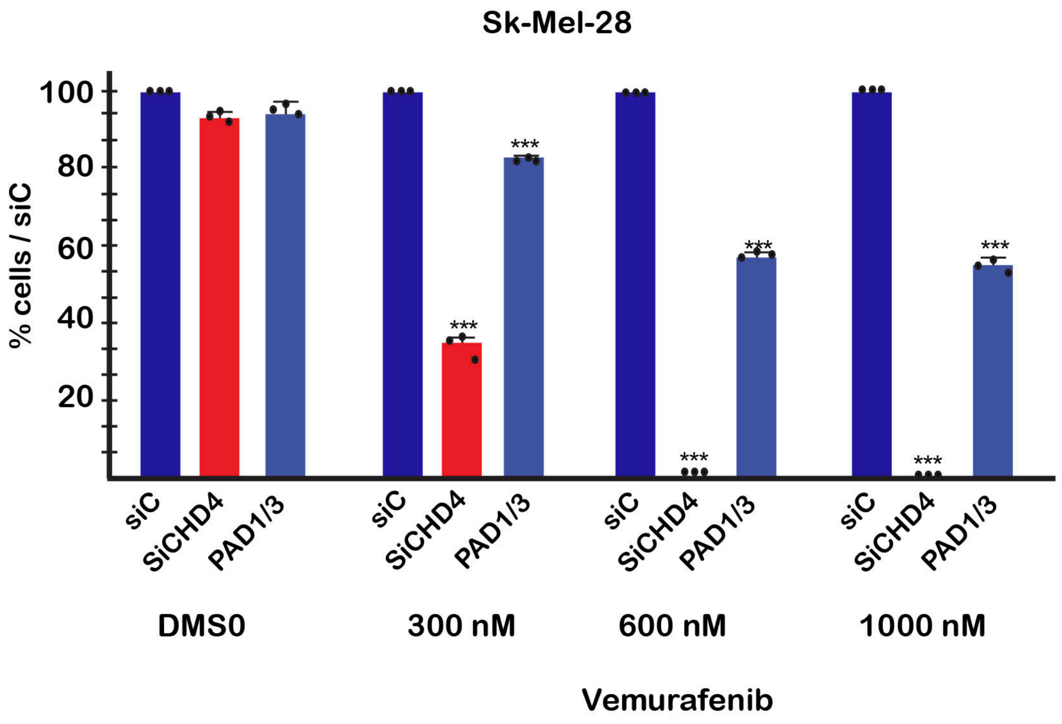

Coassolo et al. Supplementary Figure 8

Supplementary Figure 8. Citrullination modules sensitivity to BRAF inhibitor. Graph shows the % of surviving Sk-Mel28 cells compared to control 2 days after treatment with the indicated concentrations of vemurafenib. N = 3 biological replicates and unpaired t-test with two tailed P-value analyses and confidence interval 95% were performed by Prism 5. P-values: *= p<0,05; **= p<0,01; ***= *p<0,001*. Data are mean ± SEM.

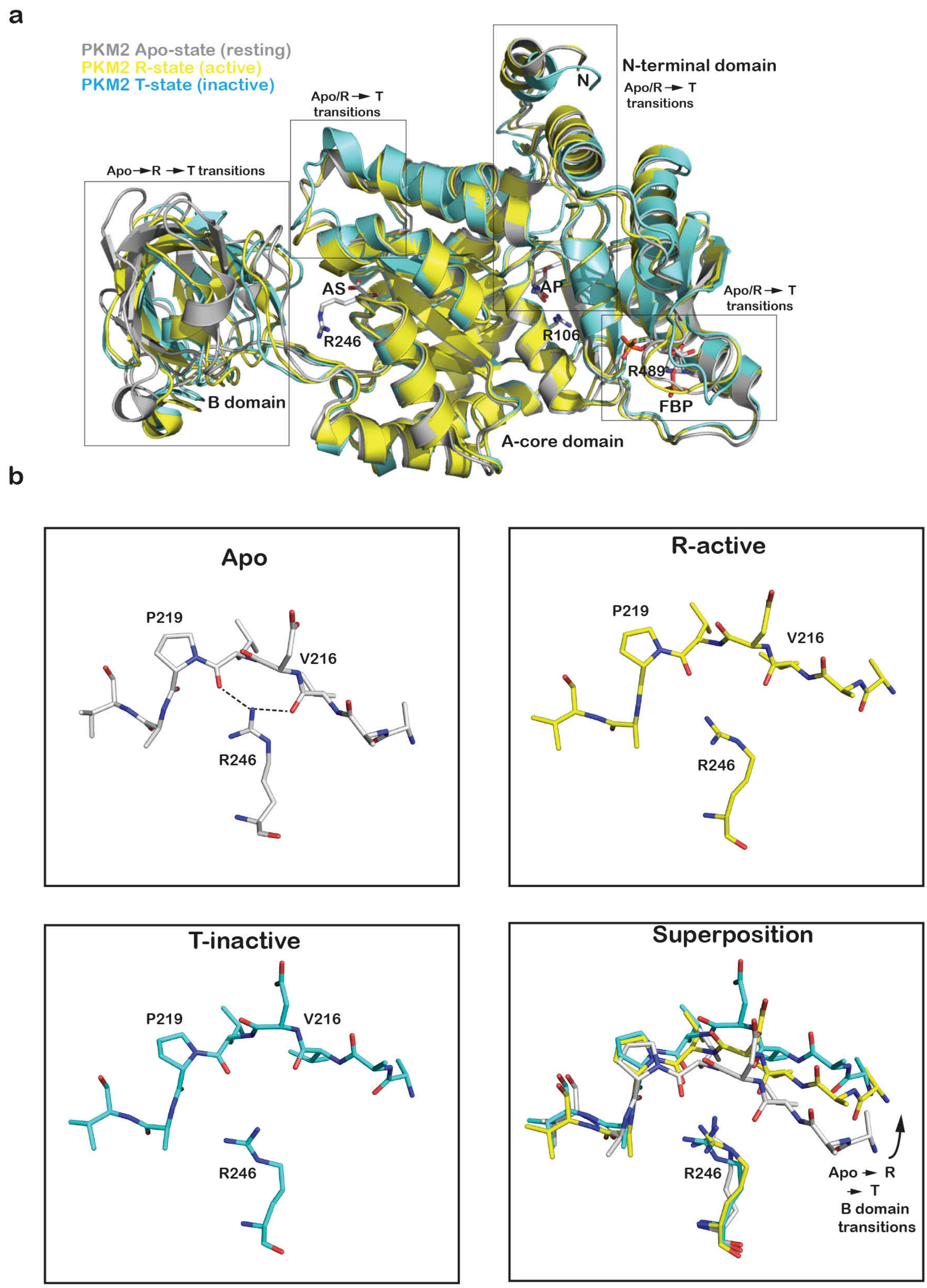

Coassolo et al., Supplementary Figure 9

Supplementary Figure 9. Locations and interactions of citrullinated arginines in PKM2. **a.** Ribbon representation of a PKM2 monomer in the apo resting state (grey; PDB 3SRH), the

active R state (yellow; PDB 6GG6 with FBP and oxalate molecules from 3SRD) and the inactive T state (cyan; PDB 6GG4). Arginine residues R106, R246 and R489, the free amino acids Serine and Phenylalanine, FBP and oxalate (surrogate of pyruvate to occupy the active site) are shown as sticks (carbon, grey; nitrogen, blue; oxygen, red; phosphorus, orange). AS, active site. AP, free amino acid binding pocket. The regions of PKM2 undergoing allosteric structural transitions between the three states are boxed. **b.** Closeup view of R246 interactions with the B domain in the Apo, R-active and T-inactive states along with a superposition of the three structures. Colour coding and representation of salt bridges/hydrogen bonds as in panel a.

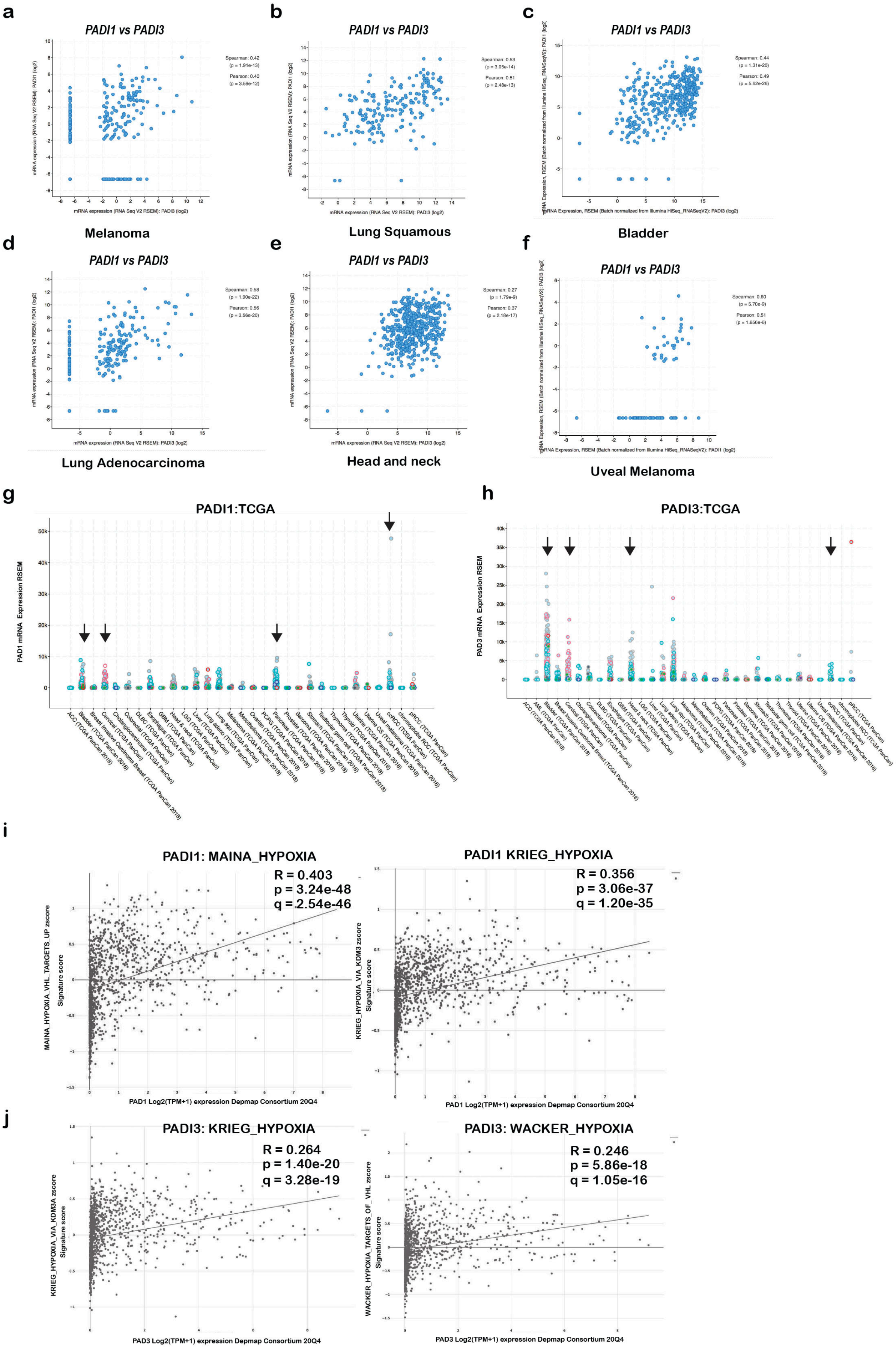

Coassolo et al., Supplementary Figure 10

Supplementary Figure 10. Analyses of *PADI1* and *PADI3* expression in TCGA datasets. **a-f**. Graphs show correlation of *PADI1* and *PADI3* expression in the indicated tumour types from the TCGA database with their Spearman and Pearson coefficients and p-values. **g-h.** *PADI1* and *PADI3* expression in the PanCancer Atlas collection of tumours from the TCGA. Arrows indicate bladder, cervical, pancreatic, head and neck and renal cell cancers. **i-j**. Correlation of *PADI1* and *PADI3* expression with the indicated Msigbd hypoxia signatures. Pearson coefficients and p-values and q-values are indicated.

Supplementary References.

1. Fontanals-Cirera, B. *et al.* Harnessing BET Inhibitor Sensitivity Reveals AMIGO2 as a Melanoma Survival Gene. *Mol Cell* **68**, 731-744 e9 (2017).
2. Laurette, P. *et al.* Transcription factor MITF and remodeller BRG1 define chromatin organisation at regulatory elements in melanoma cells. *Elife* **10.7554/eLife.06857**, (2015).
