# Supplementary material for: Citrullination of pyruvate kinase M2 by PADI1 and PADI3 regulates glycolysis and cancer cell proliferation
Source: Nat Commun. 2021 Mar 19;12:1718. doi: 10.1038/s41467-021-21960-4 (PMC7979715; doi:10.1038/s41467-021-21960-4)
Supplement: Supplementary file 3 — Description of Additional Supplementary Files [file 41467_2021_21960_MOESM3_ESM.pdf]

Description of Additional Supplementary Files

Supplementary Data 1. Summary of RNA-seq results following CHD4 silencing in 501Mel cells. Shown are gene names, description, fold change, p-value and adjusted p-value. As indicated, other pages on the spreadsheet show the ontology analyses of each gene set.

Supplementary Data 2. Proteins enriched after pan-citrulline immunoprecipitation from CHD4 silenced cells. Shown are accessions, gene names, gene descriptions, -Log P-values, differences (siCHD4-siCTRL), sum peptides scores, percentage of coverage, peptide number, PSM number, NSAF values (PSMs/protein length), unique peptide numbers, amino acid number and molecular mass.

Supplementary Data 3. A list of oligonucleotides, antibodies and resources.
