# Supplementary material for: Citrullination of pyruvate kinase M2 by PADI1 and PADI3 regulates glycolysis and cancer cell proliferation
Source: Nat Commun. 2021 Mar 19;12:1718. doi: 10.1038/s41467-021-21960-4 (PMC7979715; doi:10.1038/s41467-021-21960-4)
Supplement: Supplementary file 7 — Reporting summary [file 41467_2021_21960_MOESM7_ESM.pdf]

nature research

Corresponding author(s): DAVIDSON Irwin

Last updated by author(s): February 09 2021

Reporting Summary

Nature Research wishes to improve the reproducibility of the work that we publish. This form provides structure for consistency and transparency in reporting. For further information on Nature Research policies, see Authors & Referees and the Editorial Policy Checklist .

Statistics

For all statistical analyses, confirm that the following items are present in the figure legend, table legend, main text, or Methods section.

n/a	Confirmed	
☐	☒	The exact sample size (*n*) for each experimental group/condition, given as a discrete number and unit of measurement
☐	☒	A statement on whether measurements were taken from distinct samples or whether the same sample was measured repeatedly
☐	☒	The statistical test(s) used AND whether they are one- or two-sided<br>*Only common tests should be described solely by name; describe more complex techniques in the Methods section.*
☒	☐	A description of all covariates tested
☒	☐	A description of any assumptions or corrections, such as tests of normality and adjustment for multiple comparisons
☐	☒	A full description of the statistical parameters including central tendency (e.g. means) or other basic estimates (e.g. regression coefficient) AND variation (e.g. standard deviation) or associated estimates of uncertainty (e.g. confidence intervals)
☐	☒	For null hypothesis testing, the test statistic (e.g. *F*, *t*, *r*) with confidence intervals, effect sizes, degrees of freedom and *P* value noted<br>*Give P values as exact values whenever suitable.*
☒	☐	For Bayesian analysis, information on the choice of priors and Markov chain Monte Carlo settings
☒	☐	For hierarchical and complex designs, identification of the appropriate level for tests and full reporting of outcomes
☐	☒	Estimates of effect sizes (e.g. Cohen's *d*, Pearson's *r*), indicating how they were calculated

Our web collection on statistics for biologists contains articles on many of the points above.

Software and code

Policy information about availability of computer code

Data collection	*Provide a description of all commercial, open source and custom code used to collect the data in this study, specifying the version used OR state that no software was used.*
Data analysis	Flowjo software v 6.8.: SequestHT algorithm in Proteome Discoverer 2.4 : Perseus: Bowtie 1.2.2. : GSEA v3.0: Molecular Signatures Database v6.2. MACS2

For manuscripts utilizing custom algorithms or software that are central to the research but not yet described in published literature, software must be made available to editors/reviewers. We strongly encourage code deposition in a community repository (e.g. GitHub). See the Nature Research guidelines for submitting code & software for further information.

Data

Policy information about availability of data

All manuscripts must include a data availability statement. This statement should provide the following information, where applicable:

- Accession codes, unique identifiers, or web links for publicly available datasets
- A list of figures that have associated raw data
- A description of any restrictions on data availability

The data sets described here been deposited at GEO with the accession number GSE134850

Field-specific reporting

Please select the one below that is the best fit for your research. If you are not sure, read the appropriate sections before making your selection.

☒ Life sciences ☐ Behavioural & social sciences ☐ Ecological, evolutionary & environmental sciences

For a reference copy of the document with all sections, see nature.com/documents/nr-reporting-summary-flat.pdf

Life sciences study design

All studies must disclose on these points even when the disclosure is negative.

Sample size	N= minimum of 3 biological replicates and between 3 and 6 technical replicates were used to generate data. Data from each given experimental condition showed no significant differences indicating a sufficient sample size.
Data exclusions	None
Replication	All attempts at replications were successful
Randomization	Not relevant for this study where each sample was subjected to a specific experimental procedure.
Blinding	Blinding was not possible as the data were acquired and analyzed by the same person that performed the experimental procedure

Reporting for specific materials, systems and methods

We require information from authors about some types of materials, experimental systems and methods used in many studies. Here, indicate whether each material, system or method listed is relevant to your study. If you are not sure if a list item applies to your research, read the appropriate section before selecting a response.

Materials & experimental systems

n/a	Involved in the study	
☐	☒	Antibodies
☐	☒	Eukaryotic cell lines
☒	☐	Palaeontology
☒	☐	Animals and other organisms
☒	☐	Human research participants
☒	☐	Clinical data

Methods

n/a	Involved in the study	
☐	☒	ChIP-seq
☐	☒	Flow cytometry
☒	☐	MRI-based neuroimaging

Antibodies

Antibodies used	Pan Citrulline antibody Abcam, ab6464, CHD3 abcam ab84528, CHD4 Abcam ab72418, MITF Abcam ab3201, PADI1 Abcam ab181726, PADI3 Abcam ab172959, PKM Santa Cruz sc-365684, SOX10 Abcam, ab155279, PKM1 Cell Signaling Technology 7067T, PKM2 Cell Signaling Technology #3198S Tubulin; Abcam ab6046, HRP Goat anti mouse (GAMPO) : Jackson ImmunoResearch Laboratories ref 115-036-071; HRP Goat anti rabbit (GARPO) : Jackson ImmunoResearch Laboratories ref 111-035-144
Validation	https://www.abcam.com/citrulline-antibody-ab6464.html<br>https://www.abcam.com/products?keywords=abcam+ab84528<br>https://www.abcam.com/chd4-antibody-ab72418.html<br>https://www.abcam.com/mitf-antibody-d5-ab3201.html<br>https://www.abcam.com/padi1--pad1-antibody-ab181762.html<br>https://www.abcam.com/padi3--pad3-antibody-epr12165b-ab172959.html<br>https://www.abcam.com/sox10-antibody-epr4007-ab155279.html<br>https://www.abcam.com/beta-tubulin-antibody-loading-control-ab6046.html<br>https://www.scbt.com/fr/p/pkm-antibody-c-11<br>https://www.cellsignal.com/products/primary-antibodies/pkm1-d30g6-xp-rabbit-mab/7067<br>https://www.cellsignal.com/products/primary-antibodies/pkm2-antibody/3198<br>https://www.jacksonimmuno.com/catalog/products/115-036-071<br>https://www.jacksonimmuno.com/catalog/products/111-035-144

Eukaryotic cell lines

Policy information about cell lines

Cell line source(s)	501Mel, Sk-Mel-28, SiHa, Hela, UOK109, A498 MCF7 and WI-38 from ATCC, Hermes-3A were established and provided by Dr D Bennet London, MM117 and MM074 from Dr G. Ghanem Leuven Belgium.

October 2018

Authentication	UOK109 verified by expression of NONO-TFE3 fusion protein, A498 by presence of described somatic mutations. All other cell lines were authenticated via ATCC or the providers
Mycoplasma contamination	All lines tested negative for mycoplasma contamination
Commonly misidentified lines (See ICLAC register)	None

ChIP-seq

Data deposition

☒ Confirm that both raw and final processed data have been deposited in a public database such as GEO.

☒ Confirm that you have deposited or provided access to graph files (e.g. BED files) for the called peaks.

Data access links *May remain private before publication.*	GSE134850
Files in database submission	RNA-seq Files; siC-1, siC-2, siC-3, siCHD3-1, siCHD3-2, siCHD3-3. siC-1, siC-2, siC-3, siCHD41 sCHD4-2,siCHD4-3. ChIP-seq. SNCL47.fastq.gz Input Chromatin. SNCL49.fastq.gz CHD4 ChIP.
Genome browser session (e.g. UCSC)	http://genome-euro.ucsc.edu/s/Irwin/CHD4%2DMelanoma

Methodology

Replicates	1
Sequencing depth	SNCL 47 Total reads 66 482 761, unique reads 55 232 825<br>SNCL 49 Total reads 72 454 814, unique reads 61 118 955<br>50bp single end reads
Antibodies	CHD4 abcam ab72418; Lot:GR271300-12
Peak calling parameters	Sequenced reads were mapped to the Homo sapiens genome assembly hg19 using Bowtie with the following arguments: -m 1 --strata --best -y -S -l 40 -p 2.<br>Peak calling with MACS. Parameters: -q 0.01 --broad --nomodel --extsize 151
Data quality	60125 peaks with FDR 1%
Software	Sequenced reads were mapped to the Homo sapiens genome assembly hg19 using Bowtie with the following arguments: -m 1 --strata --best -y -S -l 40 -p 2. After sequencing, peak detection was performed using the MACS software

Flow Cytometry

Plots

Confirm that:

☒ The axis labels state the marker and fluorochrome used (e.g. CD4-FITC).

☒ The axis scales are clearly visible. Include numbers along axes only for bottom left plot of group (a 'group' is an analysis of identical markers).

☒ All plots are contour plots with outliers or pseudocolor plots.

☒ A numerical value for number of cells or percentage (with statistics) is provided.

Methodology

Sample preparation	Cells were grown as described in each experiment and labelled with CellTRace Violet at day of transfection and cultured for an additional 72 hours before harvesting and flow cytometry. Cells were transfected with siRNA, harvested after 72 hours and stained with Annexin V- FITC (Fluorescein isothiocyanate) and Propidium Iodide before flow cytometry.
Instrument	LSRII Fortessa (BD Biosciences)
Software	Flowjo software v 6.8.
Cell population abundance	N/A

Gating strategy	For Cell Trace Violet incorporation, cells were first gated by FSC-A/SSC-A to separate live cells from dead cells and debris. Live cell fractions (80-95%) were then gated FSC-A/FSC-H to identify single cells and remove doublets with abnormal FSC-A/FSC-H ratio. Gated single cell fluorescence of Brilliant Violet 421 was then assessed and quantified.<br>For apoptosis, cells were gated using Propidium Iodide and Annexin V-FITC.

☒ Tick this box to confirm that a figure exemplifying the gating strategy is provided in the Supplementary Information.
